# Effect of occupational therapy on the occurrence of delirium in critically ill patients: a systematic review and meta-analysis

**DOI:** 10.3389/fneur.2024.1391993

**Published:** 2024-07-22

**Authors:** Jun Zhao, Kaipeng Fan, Suqin Zheng, Guangyao Xie, Xuekang Niu, Jinkuo Pang, Huihuang Zhang, Xin Wu, Jiayang Qu

**Affiliations:** ^1^Rehabilitation Assessment and Treatment Center, The Third Affiliated Hospital of Zhejiang Chinese Medical University, Hangzhou, Zhejiang, China; ^2^School of Rehabilitation Medicine, Zhejiang Chinese Medical University, Hangzhou, Zhejiang, China; ^3^Department of Rehabilitation, Hangzhou Seventh People's Hospital, Hangzhou, Zhejiang, China

**Keywords:** occupational therapy, delirium, critically ill patients, rehabilitation therapy, safety

## Abstract

**Aim:**

Delirium poses a major challenge to global health care, yet there is currently a dearth of single effective interventions or medications. Particularly, addressing delirium induced by critical illness is a complex process. Occupational therapy is considered to have a high potential for use in the prevention of delirium, as it involves both cognitive training and training in ADL. To comprehensively analyze the effect of occupational therapy on delirium prevention, we evaluated the effects of occupational therapy vs. standard non-pharmacological prevention on incidence and duration of delirium, clinical outcomes and rehabilitation outcomes in critically ill patients.

**Methods:**

The data sources, including PubMed/Medline, Web of Science, EMBASE, and Cochrane Library, were comprehensively searched from their inception until 15 October 2023. Following the PICOS principle, a systematic screening of literature was conducted to identify relevant studies. Subsequently, the quality assessment was performed to evaluate the risk of bias in the included literature. Finally, outcome measures from each study were extracted and comprehensive analysis was conducted using Review Manager 5.4.

**Results:**

A total of four clinical trials met the selection criteria. The pooled analysis indicated no significant difference in the incidence and duration of delirium between the OT group and standard non-pharmacological interventions. A comprehensive analysis of clinical outcomes revealed that OT did not significantly reduce the length of hospital stay or ICU stay. Meanwhile, there was no significant difference in mortality rates between the two groups. It is noteworthy that although grip strength levels did not exhibit significant improvement following OT intervention, there were obvious enhancements observed in ADL and MMSE scores.

**Conclusion:**

Although occupational therapy may not be the most effective in preventing delirium, it has been shown to significantly improve ADL and cognitive function among critically ill patients. Therefore, we contend that occupational therapy is a valuable component of a comprehensive multidisciplinary approach to managing delirium. In the future, high-quality researches are warranted to optimize the implementation of occupational therapy interventions for delirium prevention and further enhance their benefits for patients.

## 1 Introduction

Delirium, as defined in the diagnostic and statistical manual of mental disorders 5 (DSM-5), manifests as attention disorders and cognitive impairments that develop over a short period of time and tend to fluctuate in severity throughout the day (1). Despite delirium was first described over 2,500 years ago (2), its pathophysiology remains inadequately understood, and lacking a singular efficacious intervention or pharmaceutical agent for its treatment (3, 4). Delirium poses a significant global healthcare challenge, with reported prevalence rates of approximately 15.2% in geriatric emergency departments globally (5). More than half of all patients in a modern intensive care unit (ICU) will develop delirium at some point during their admission (6). In the United States, more than 2.6 million adults aged 65 years and older develop delirium each year (7).

Most notably, delirium represents an augmented risk of long-term cognitive impairment (8), prolonged periods of mechanical ventilation and hospital stays (9, 10), and escalated medical costs (11). Consequently, it becomes imperative to identify at-risk individuals and promptly recognize and address the causative factors precipitating delirium. Additionally, implementing early preventive measures among high-risk patients may offer an additional approach to reducing the incidence of delirium. Despite increasing evidence and recommendations for the use of drugs such as dexmedetomidine, remifentanil, ketamine, and others in the management of pain and delirium in critically ill adults (12), pharmacologic prophylaxis is only indicated for patients at high risk for delirium and not generally recommended (13).

In this context, non-pharmacological interventions may be another avenue to prevent the development of delirium in critically ill patients (14). Occupational therapy (OT), as a pivotal component of critical care rehabilitation, which includes cognitive function training, environmental modification, family intervention and social psychological support and other methods are considered to be one of the non-pharmacological interventions for delirium prevention. However, the effectiveness of OT in delirium prevention remains inadequately supported by high-quality studies, which cannot provide sufficient guidance for clinical application. The systematic review by Cupka et al. (15) considered multicomponent, bundled interventions to be more effective in managing ICU delirium than approaches using single intervention factors, but comparative studies should be conducted to determine the importance of specific bundle elements. Costigan et al. (16) conducted a scoping review of 221 ICU occupational therapy related literature and concluded that the role of occupational therapists in ICU rehabilitation has not been well established. Therefore, our goal is to evaluate the effectiveness of OT vs. standard therapy or other non-pharmacological interventions on the incidence, duration, and other clinical outcomes of delirium in critically ill patients.

## 2 Methods

Positionality statement: The authors of this study consist of occupational therapists, speech therapists, and doctors. The authors developed an interest in the management of delirium within the critical care rehabilitation practice, prompting them to delve into this subject matter. Formal ethical approval was not required for the literature review as all data collected are secondary data. The detailed protocol was registered in the PROSPERO (CRD42023472589, https://www.crd.york.ac.uk/PROSPERO/). The preferred reporting checklist (PRISMA) of systematic reviews and meta-analysis were used to guide this study (Supplementary material 1).

### 2.1 Inclusion criteria

(a) Population: patients 18 years of age and older who were hospitalized for more than 24 h in the ICU, regardless of region, gender or race; (b) Intervention: OT combined with standard non-pharmacological prevention (nPP) or standard care interventions; (c) Comparisons: Standard measures include time and place reorientation, early mobilization, correction of sensory deficits, environmental management, reduction of medication, etc.; (d) Outcomes: the indicators are the number of patients presenting with delirium and duration, length of hospital stay (LOS), ICU-LOS, mortality, score of Mini-Mental State Examination (MMSE), hand grip and score of Activities of Daily Living (ADL); (e) Study Types: Randomized Controlled Trials (RCTs).

### 2.2 Exclusion criteria

Reports, reviews, abstracts, trials and letters with duplicate, incomplete and unavailable data were excluded. In addition, studies unrelated to the topic of OT prevention of delirium (for example, the patients included in the study had developed delirium or the intervention group had not implemented OT) are excluded.

### 2.3 Data sources

The following English databases were searched from the inceptions to 15 October 2023: PubMed/Medline, Web of Science, EMBASE, Cochrane library. The MeSH and keywords search terms included Occupational Therapy, Occupational Therapies, Ergotherapy, Ergotherapies, Delirium, Subacute Delirium. A detailed illustration of search strategies is available in Supplementary material 2.

### 2.4 Data extraction and quality assessment

Two independent reviewers evaluated the retrieved studies for inclusion and assessed the methodological quality of included studies. Elements extracted included study characteristics (author, country, publication year and design), participant characteristics (age range, Inclusion and exclusion criteria), intervention details and outcome measures. The risk of bias was assessed using Review Manager 5.3 software. In instances of disagreement between two data extractors, the third examiner intervenes to facilitate resolution.

### 2.5 Data analysis

Data entry and analysis were performed using Review Manager 5.3 software. The data required for meta-analysis was directly extracted from the original literature or indirectly calculated on the basis of the original data through the conversion tool^1^ developed by Chinese scholars [For example, the quartiles in the study were converted to standard deviation (SD)]. The random effects model and its index standardized mean difference (SMD) were used in the combined analysis of continuous variables to reduce the impact of the differences brought by the transformed data on the combined analysis, while risk ratio (RR) was used to compare binary variables (mortality and number of delirium). All results obtained were reported with 95% confidence intervals (CI). Heterogeneity among studies was determined by Q test and I^2^ statistics [Cochrane book 9.5.2 Identifying and measuring heterogeneity, 0%–40%: might not be important; 30%–60%: may represent moderate heterogeneity*; 50%–90%: may represent substantial heterogeneity*; 75%–100%: considerable heterogeneity* (17)]. With substantial heterogeneity, sensitivity analysis or subgroup analysis was used to detect the source of heterogeneity; if the source of heterogeneity cannot be found, a descriptive analysis was conducted. Due to the small number of studies included in this paper, it is not suitable to use funnel plot to evaluate publication bias.

## 3 Results

### 3.1 Results of the search

**Figure 1:**
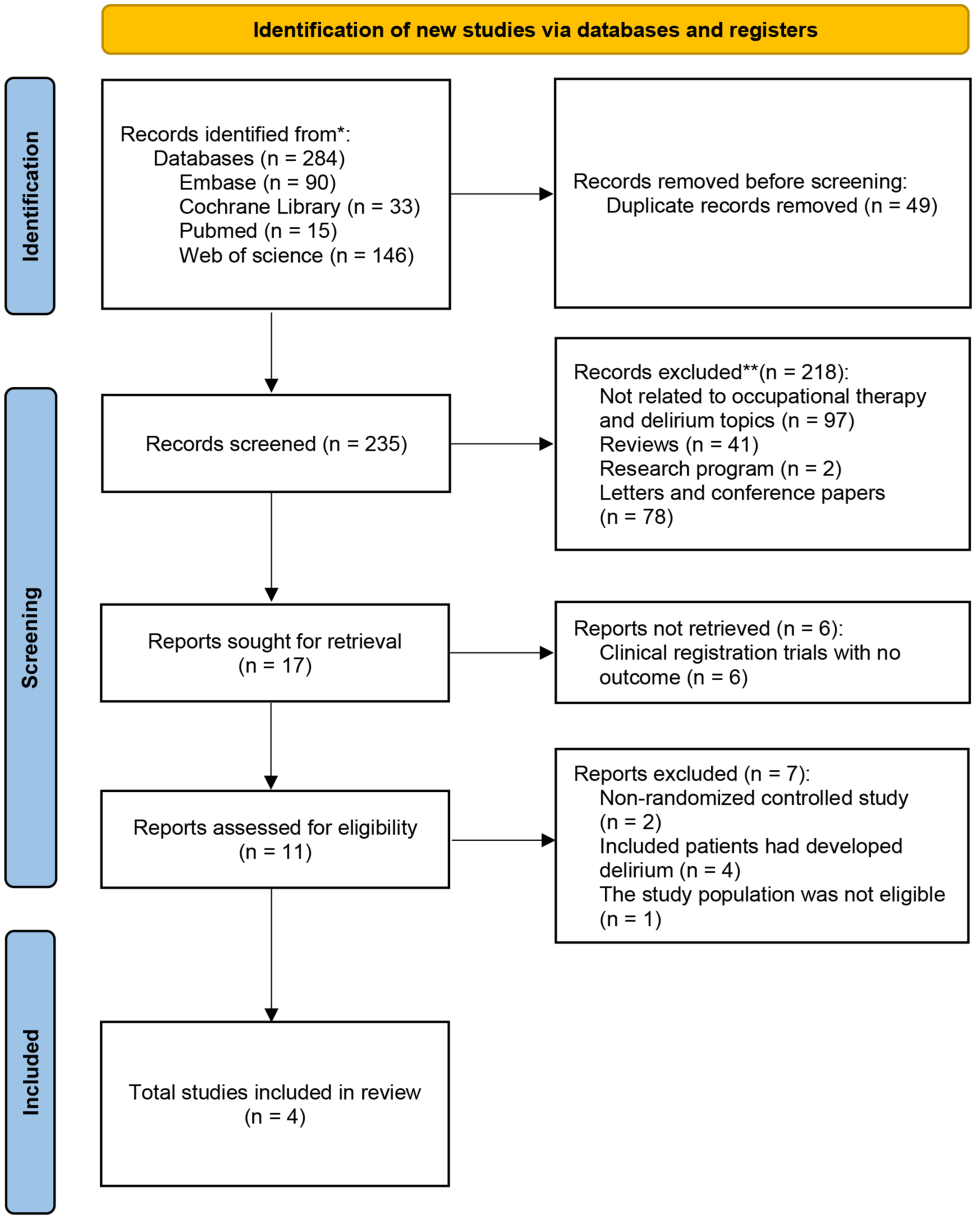
Literature selection and inclusion process.

A total of 284 articles were retrieved from 4 databases: Web of Science (*n* = 146), PubMed/MEDLINE (*n* = 15), Cochrane (*n* = 33), and Embase (*n* = 90). After removing duplicates using Endnote software and manual screening, 235 records were included for further review (Figure 1). Subsequently, 218 records did not meet our inclusion criteria and were excluded, as were six experimental registers with unpublished results. Upon further examination of the original articles, we excluded two non-RCT studies, four studies that included patients already exhibiting symptoms of delirium, and one study that did not meet the inclusion population criteria. Finally, four studies were included in our meta-analysis.

### 3.2 Characteristics of the studies

The characteristics of the included studies are presented in Table 1. The meta-analysis included four RCT studies, with 2 from the United States, 1 from Chile, and 1 from Canada. All the studies included patients in the ICU, with an average age ranging from 63 to 71 years. Deemer et al. (20) was the only study to use the Intensive Care Delirium Screening Checklist (ICDSC) for delirium detection, while the remaining studies used the Confusion Assessment Method (CAM). The sample sizes for the final analysis ranged from 30 to 130 across all studies, which were published between 2009 and 2023.

**Table 1:** Summary of clinical studies on prevention of delirium by occupational therapy.

Study	Country	Trial registration information	Design	Aggregate/analyzed	Therapy			Control			Main Outcome Measures	Diagnostic criteria for delirium
					Delirious number/Sample Size	Delirium duration	Age, y	Delirious number/Sample Size	delirium duration	Age, y		
Alvarez 2017 (18)	Chile	/	Double blinding RCT	140/130	2/65	/	71 [63–78.5]	14/65	/	68 [63–75.5]	Duration, incidence and severity of delirium, ICU-LOS, LOS, Grip strength, FIM, MMSE	CAM
Brummel 2014 (19)	United States of America	NCT01270269	Single-blind RCT	65/30	_/18	3.0 [1–24.5]	62 [54–69]	_/12	2.0 [1.0–17.2]	60 [51–69]	Days free of delirium and coma, days free of mechanical ventilation, MMSE, TUG, Katz Activities of Daily Living questionnaire, FAQ, EQ-5D VAS	CAM
Deemer 2023 (20)	Canada	NCT03604809	Single-blind RCT	70/69	17/32	1 [0–2]	63 [56–71]	19/37	1 [0–3]	53 [43–64]	Delirium prevalence and duration, ICU-LOS, LOS, ACE, FSS-ICU, QoL, mortality	ICDSC
Schweickert 2009 (21)	United States of America	NCT00322010	RCT	104/104	_/49	2.0 [0.0–6.0]	57.7 [36.3–69.1]	_/55	4.0 [2.0–8.0]	54.4 [46.5–66.4]	ICU-LOS, LOS, Barthel Index score, Hand grip, mortality	CAM

RCT, randomized controlled trial; ICU, intensive care unit; IQCODE, Informant Questionnaire on Cognitive Decline in the Elderly; Katz ADL, Katz Activities of Daily Living questionnaire; ACE, adapted cognitive exam; FSS-ICU, Functional Status Score for ICU; ICDSC10, intensive care delirium screening checklist; IQR, interquartile range; LOS, length of stay; QOL, quality of life; BADL, Basic activities of daily living; POD, postoperative delirium; PODS, subsyndromal POD; TUG, Timed Up-and-Go Test; FAQ, Functional Activities Questionnaire; EQ-5D, European Quality of Life-5 Dimensions Visual Analog Scale.

### 3.3 Risk assessment of bias

**Figure 2:**
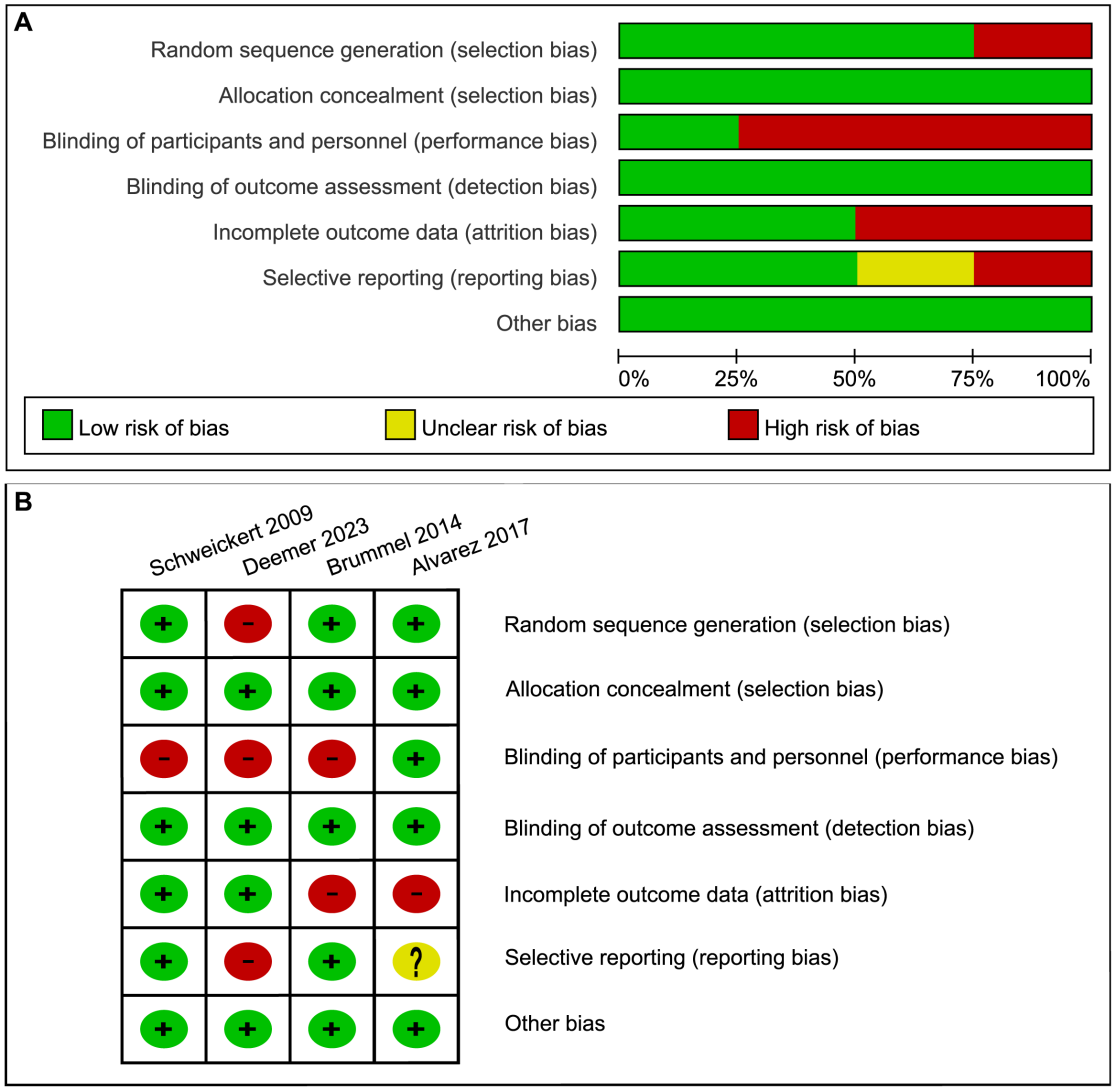
Risk of bias graph: **(A)** review authors’ judgments about each risk of bias item presented as percentages across all included studies. **(B)** Review authors’ judgments about each risk of bias item for each included study.

As all four studies analyzed were RCTs, the risk of bias related to randomization process and allocation concealment was low. Although blinding participants in occupational therapy (OT) studies is challenging given the nature of the intervention, we still strictly ruled that three studies (19–21) that did not blind participants were at high risk for performance bias. Additionally, two studies (18, 19) were considered high-risk in terms of attrition bias, as they experienced a loss in the number of participants available for data analysis compared to the initial baseline. Furthermore, Deemer et al. (20) reported that some prognostic markers were not achieved in certain participants due to illness severity, death, transfer to another ICU, or altered level of consciousness, while Alvarez et al. (18) did not provide information on clinical registration. These factors leading to a high risk of selective reporting bias for the Deemer 2023 and an unclear risk for the Alvarez 2017 (Figure 2).

### 3.4 Meta-analysis

A total of four eligible articles were included in the meta-analysis. The primary indicators encompassed the incidence and duration of delirium. Clinical outcomes included the LOS, ICU-LOS, and mortality rates. Furthermore, rehabilitation outcomes comprised the MMSE, ADL and hand grip strength.

#### 3.4.1 Primary indicators

**Figure 3:**
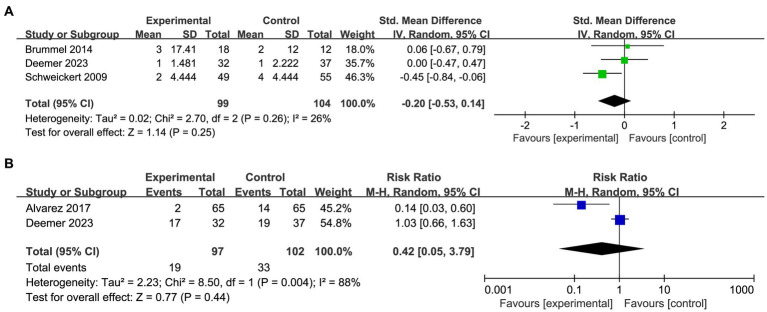
Forest plot of primary indicators: **(A)** pooled results of delirium duration. **(B)** Pooled results of the number of delirious patients.

Three studies reported the duration of delirium, including 99 patients in the trial group and 104 patients in the control group (Figure 3A). Although the data indicated a slightly shorter duration of delirium in patients receiving OT compared to the control group, there was no statistically significant difference between the two groups (SMD: −0.20; 95%CI [−0.53, 0.14]; *p* = 0.25; heterogeneity test *p* = 0.26; I^2^ = 26%). There was also no significant difference observed in the incidence of delirium cases among the two groups (RR: 0.42; 95%CI [0.05, 3.79]; *p* = 0.44; heterogeneity test *p* = 0.004; I^2^ = 88%; Figure 3B).

#### 3.4.2 Clinical outcome indicators

**Figure 4:**
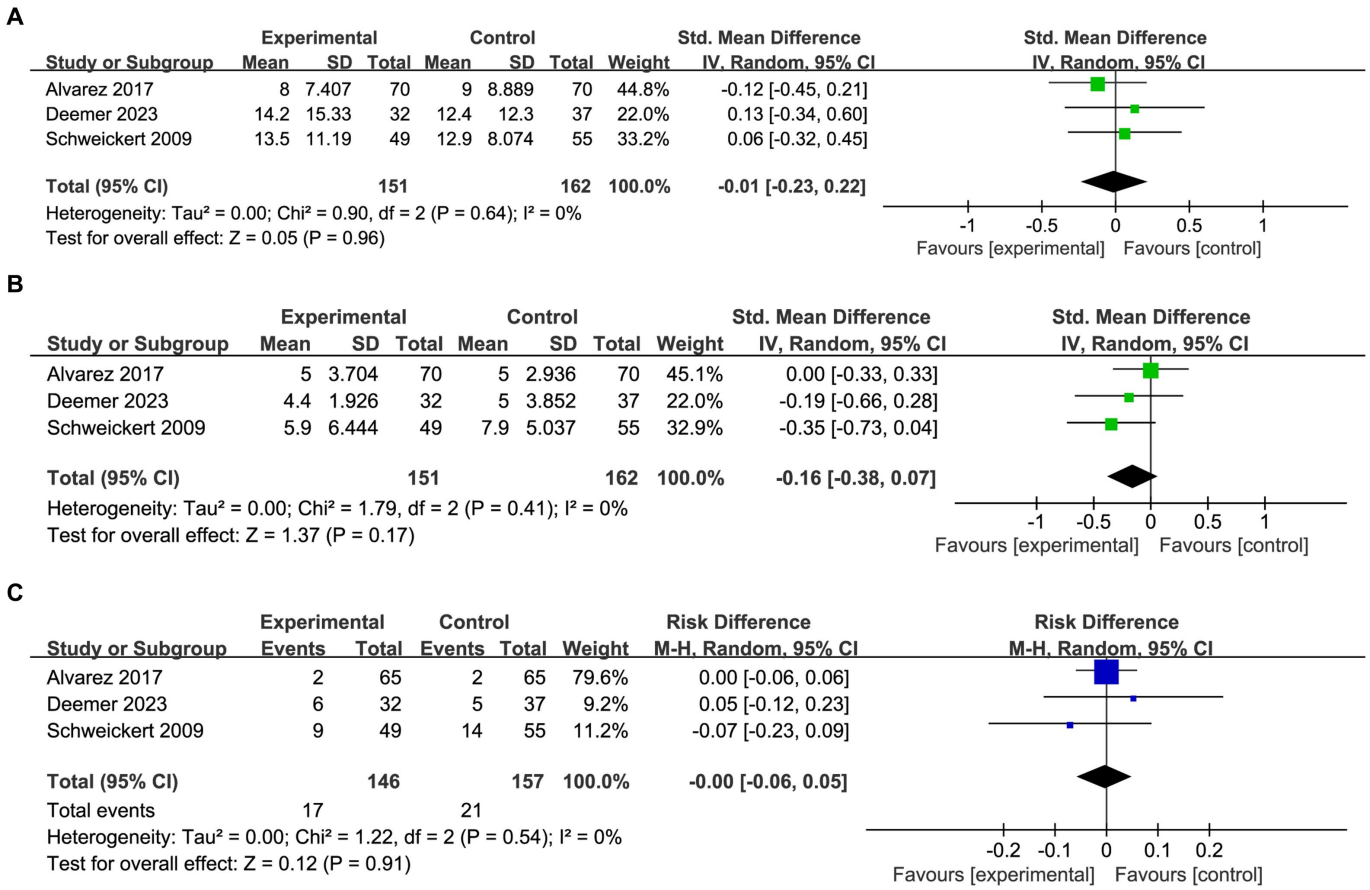
Forest plot of clinical outcome indicators: **(A)** pooled results of LOS. **(B)** Pooled results of ICU-LOS. **(C)** Pooled results of mortality.

The meta-analysis included three studies (*n* = 313 participants) investigating the impact of OT on LOS in critically ill patients (Figure 4A). The pooled analysis demonstrated that the implementation of OT did not result in a shorter LOS for the experimental group compared to the control group (SMD: −0.01; 95%CI [−0.23, 0.22]; *p* = 0.96; heterogeneity test *p* = 0.64; I^2^ = 0%). Three studies included in the analysis collected data on ICU-LOS (Figure 4B). The pooled analysis indicated a slightly shorter ICU-LOS for patients who received OT compared to those in the control group, but this difference was not statistically significant (SMD: −0.16; 95%CI [−0.38, 0.07]; *p* = 0.17; heterogeneity test *p* = 0.41; I^2^ = 0%). To assess the safety of OT in critically ill patients, a meta-analysis regarding mortality was conducted (Figure 4C). The results revealed no statistically significant difference in mortality between the two groups (RD: −0.00; 95%CI [−0.06, 0.05]; *p* = 0.91; heterogeneity test *p* = 0.54; I^2^ = 0%), thereby indicating the safety of OT in critically ill patients.

#### 3.4.3 Rehabilitation outcome indicators

**Figure 5:**
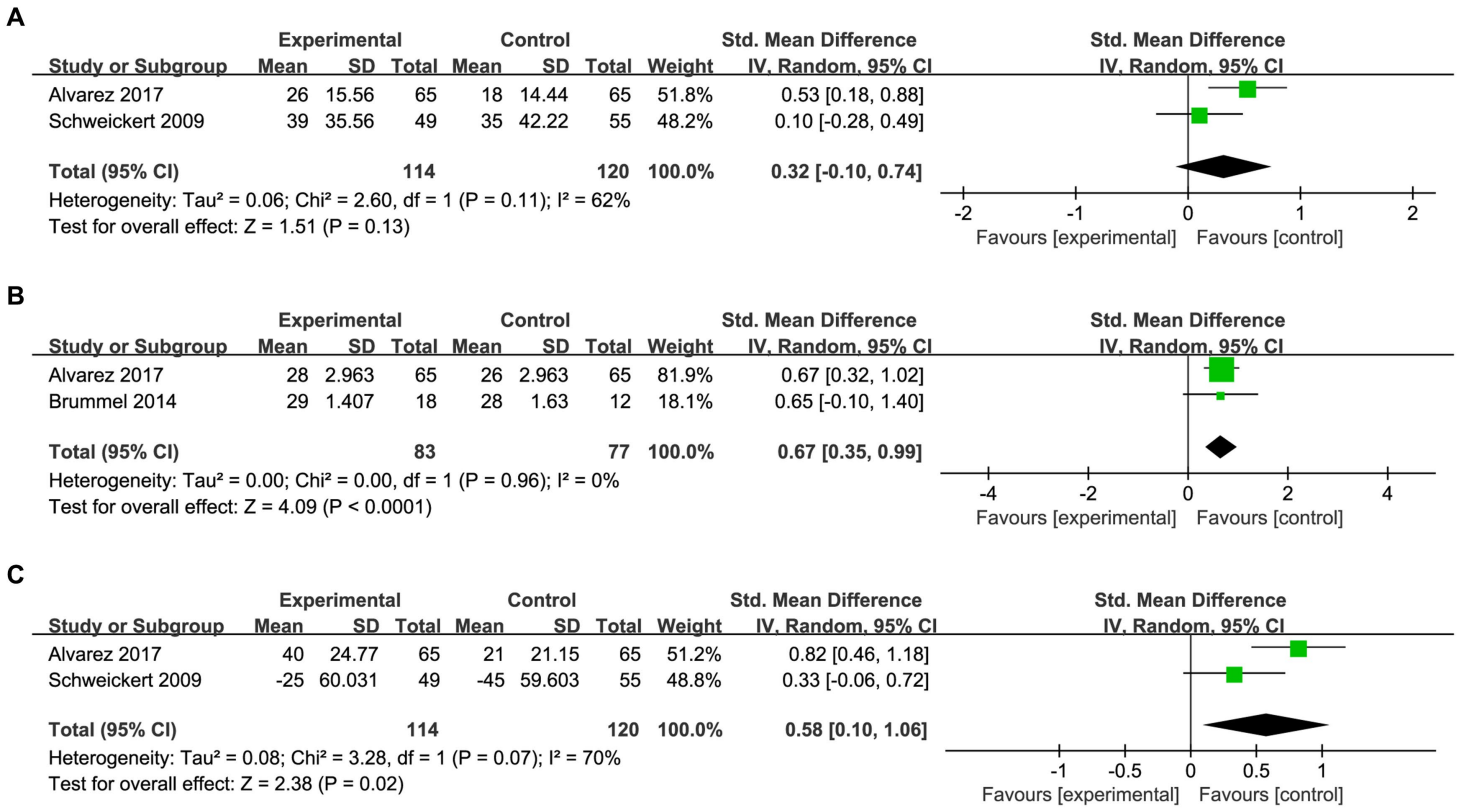
Forest plot of rehabilitation outcome indicators: **(A)** pooled results of dominant hand grip. **(B)** Pooled results of the MMSE scores. **(C)** Pooled results of the ADL scores.

The comprehensive analysis of dominant hand grip in patients was conducted based on two studies (Figure 5A). The data indicated that there was no significant improvement in grip strength observed among patients in the occupational therapy (OT) group compared to the control group (SMD: 0.32; 95%CI [−0.10, 0.74]; *p* = 0.13; heterogeneity test *p* = 0.11; I^2^ = 62%). The MMSE score was reported in two studies, encompassing 83 patients in the OT group and 77 patients in the control group (Figure 5B). Interestingly, the pooled analysis revealed a significant enhancement of MMSE scores among critically ill patients following OT intervention compared to the control group (SMD: 0.67; 95%CI [0.35, 0.99]; *p* < 0.0001; heterogeneity test *p* = 0.96; I^2^ = 0%). The motor component of the Functional Independence Measure (FIM) scale was combined with the Barthel Index to assess patients’ ADL (Figure 5C). The findings demonstrated that occupational therapy significantly enhanced ADL scores among critically ill patients (SMD: 0.58; 95%CI [0.10, 1.06]; *p* = 0.02; heterogeneity test *p* = 0.07; I^2^ = 70%).

## 4 Discussion

Delirium is one of the major concerns in the treatment of critically ill patients and necessitates a multidisciplinary approach that integrates intricate non-pharmacological interventions (22). As integral members of the rehabilitation team, occupational therapists play a pivotal role in critical care rehabilitation (23), as well as in the prevention and management of delirium. Although OT is considered promising in the prevention of delirium, its definite preventive effect still requires systematic evidence-based research and rigorous evaluation.

We included four studies that evaluated the efficacy of occupational therapy compared with standard care or other non-pharmacological interventions on delirium incidence and duration, hospital mortality, cognitive function, ability to perform activities of daily living, and grip strength. Our findings indicate that OT did not yield significant reductions in delirium duration, delirium rate, LOS, and ICU-LOS compared to other non-pharmacological interventions. However, encouraging results were observed in terms of rehabilitation outcomes. Although the grip strength of critically ill patients did not improve due to the OT intervention, both their MMSE scores and ADL scores after receiving the OT intervention showed significant improvement. These findings highlight the beneficial effects of OT in enhancing cognitive function and promoting independence in daily activities. Furthermore, our study provides evidence supporting the safety profile of OT interventions within this clinical context.

Although the results of the meta-analysis suggested that occupational therapy did not demonstrate significant effectiveness in preventing delirium, this should not be a reason to exclude occupational therapy from non-pharmacologic approaches for delirium prevention. The findings support the notion that critically ill patients can obtain the improvement of daily living ability and cognitive ability from OT, with no evidence indicating adverse outcomes associated with such interventions. Therefore, we consider that OT is a meaningful component of the multidisciplinary integrated interventions for delirium. However, it is imperative to investigate the underlying reasons for the lack of significant effect of OT in the prevention of delirium, in order to maximize their benefits for critically ill patients.

The non-pharmacological preventive measures implemented in the control groups of each study may have elicited a maximal preventive effect, thereby rendering it improbable for additional OT measures to yield greater benefits than the existing treatment strategies. Common non-pharmacological strategies for delirium prevention encompass reorientation protocol, early mobilization, sensory deficit correction, environmental management, sleep protocol, hydration protocol, reduction of medication (18, 24–27), some of which overlap with OT intervention content in included studies. This may account for the lack of significant difference in delirium prevention between the OT group and the control group. Consequently, updating the current OT scheme is necessary to optimize its efficacy in preventing delirium. We discovered that the OT protocols employed in the included studies primarily focused on orientation, sensory stimulation, stress management, and other limited treatment contents. This phenomenon may be attributed to the poor physical condition of critically ill patients. However, factors such as the individual’s developmental stage, habits, roles, lifestyle preferences, and environment cannot be disregarded when formulating occupational therapy plan (28). Ikiugu et al. (29) concluded that occupational therapy practitioners could enhance therapeutic outcomes for their clients by effectively incorporating a combination of meaningful and psychologically rewarding occupations as intervention strategies. Schaller et al. (30) suggested that early, goal-directed activities improved patient mobilization throughout ICU admission, shortened ICU-LOS, and improved patients’ functional mobility at hospital discharge. An increasing number of hospitals are recognizing the benefits of family involvement in overall ICU patient care and are beginning to explore the adoption of unrestricted visitation policies (31). Therefore, considering the requirements of patients and their families, designing meaningful occupational activities, and involving family members in the implementation of OT may serve as an additional approach to enhance the effectiveness of OT in delirium prevention.

It’s worth noting that another component of rehabilitation medicine, physical therapy (PT), was used as a common intervention for both intervention and control groups in 3 studies (18, 19, 21). Numerous studies have demonstrated the cognitive benefits of physical activity (32–35), including improved attention, learning, memory, general intellectual functioning, executive functioning, and mental processing speed, and reduced depression and anxiety. These benefits may be attributed to specific mechanisms such as angiogenesis, neurogenesis, and the release of neurotrophins that enhance plasticity (36, 37). Chen et al. (38) reported that PT improved functional status and survival in patients undergoing prolonged mechanical ventilation. The cognitive domain scores of the FIM increased significantly in the PT group compared with the control group at 6 months after enrollment. Therefore, it is plausible that OT and PT exert distinct yet synergistic effects in delirium prevention.

Furthermore, the included studies exhibited substantial heterogeneity in terms of treatment intensity, frequency, and content, potentially impeding the optimization of preventive effects. In most of the included studies, OT regimens were conducted twice a day, but the duration of a single session ranged from 20 to 40 min. Several studies (39–42) have reported that early rehabilitation incorporating OT could effectively prevent muscle weakness, enhance exercise capacity, and improve ADL in critically ill patients. However, a consensus regarding the optimal dosage of rehabilitation therapy implementation has not yet been reached. The Japanese Clinical Practice Guidelines for Rehabilitation in Critically Ill Patients 2023 suggested the implementation of multiple daily rehabilitation sessions for critically ill patients (43), although the quality of evidence supporting this recommendation is extremely low. A guideline on the management of adult ICU patients recommends performing rehabilitation/mobilization interventions over usual care or over similar interventions with a reduced duration, reduced frequency, or later onset (44). Existing clinical practice guidelines and systematic reviews on traumatic brain injury (TBI) acknowledge the benefits of rehabilitation consisting of PT and OT, while emphasizing the need for further research to identify optimal interventions in this domain (45–47). Several stroke rehabilitation guidelines (48, 49) mention that stroke survivors should receive scheduled rehabilitation treatment as frequently as possible and utilize group cycle therapy to extend the duration of such treatment. Additionally, stroke survivors are encouraged to continue autonomous, independent practice or semi-supervised and supportive practice with family and friends outside of scheduled treatment sessions. We contend that these recommendations are equally applicable to the rehabilitation of critically ill patients. However, it is important to note that critically ill patients may exhibit poorer physical condition compared to those in the sequelae stage of stroke, which could potentially impact the implementation of OT. We believe that the specific amount of treatment (number of treatments per day, duration of each treatment session, and metabolic equivalent consumed by treatment activities) should be tailored to individual needs. Therefore, important directions for future research include understanding differences in patient outcomes according to the type of intervention as well as the timing, frequency, duration, and intensity of the intervention.

Another overlooked detail that impacts the effectiveness of OT interventions is the choice of screening tool for delirium. As mentioned earlier, three out of four studies included in this analysis utilized CAM-ICU as a screening tool, while the remaining study employed ICDSC. Although both CAM-ICU and ICDSC demonstrate satisfactory performance in detecting delirium among critically ill patients (50), it should be noted that CAM-ICU’s accuracy may be influenced by sedation levels, whereas the sensitivity and specificity of ICDSC are comparatively lower than those of CAM-ICU (51). Toro et al. (52) revealed a sensitivity of 79.4% and a specificity of 97.9% for CAM-ICU, but a subgroup analysis of mechanically ventilated patients showed an increase in sensitivity accompanied by a decrease in specificity (sensitivity: 92.9%, specificity: 86.7%). Furthermore, it has been observed that both CAM-ICU and ICDSC have worse sensitivity when testing patients with low activity delirium (53). Dure to the non-dichotomous nature of ICDSC, subsyndromal delirium can be diagnosed, enabling earlier intervention in high-risk patients. Consequently, a comprehensive utilization of delirium screening tools and timely intervention in at-risk individuals may yield enhanced benefits.

We synthesized the data from the included studies to draw conclusions and discussed areas that required further improvement in utilizing OT for preventing delirium. Undoubtedly, significant limitations remain in this systematic review. The four included studies exhibited significant heterogeneity in terms of intervention content, implementation methods, and outcome measures. The reporting of outcome indicators in various formats posed challenges in presenting data and evaluating results effectively. We had to transform the data, which resulted in unavoidable deviations from the original dataset. Therefore, for a precise and comprehensive analysis of the effectiveness of OT in preventing delirium, it is crucial to establish standardized core outcome measurement sets and reporting forms for future trials.

## 5 Conclusion

Overall, our findings demonstrate that OT effectively improves ADL and cognitive function in critically ill patients, while also exhibiting a comparable safety profile to standard non-pharmacological interventions. Despite the evidence for its superiority in the prevention of delirium is still inconclusive, we still recommend it as one of the multidisciplinary interventions for delirium prevention. We hypothesize that the concurrent use of other non-pharmacological interventions may overshadow the specific impact of OT on delirium prevention due to overlapping intervention contents. Therefore, future high-quality clinical studies should focus on developing personalized programs and innovative measures tailored to individual patient activity levels, thereby maximizing the unique role of OT in preventing delirium.

## Author contributions

JZ: Data curation, Methodology, Software, Visualization, Writing – original draft, Writing – review & editing. KF: Data curation, Formal analysis, Writing – original draft, Writing – review & editing. SZ: Data curation, Formal analysis, Software, Visualization, Writing – review & editing. GX: Formal analysis, Software, Supervision, Writing – review & editing. XN: Methodology, Project administration, Supervision, Visualization, Writing – review & editing. JP: Formal analysis, Writing – original draft. HZ: Data curation, Visualization, Writing – review & editing. XW: Formal analysis, Writing – original draft. JQ: Conceptualization, Formal analysis, Methodology, Resources, Supervision, Writing – original draft, Writing – review & editing.

## Footnotes

^1^ https://www.yxzlb.com/forum.php?mod=viewthread&tid=3679&page=1#pid9919
