# Supplementary material for: Effect of occupational therapy on the occurrence of delirium in critically ill patients: a systematic review and meta-analysis
Source: Front Neurol. 2024 Jul 22;15:1391993. doi: 10.3389/fneur.2024.1391993 (PMC11298357; doi:10.3389/fneur.2024.1391993)
Supplement: Supplementary file 2 [file Data_Sheet_2.docx]

**search strategies**

**Pubmed**

(("Occupational Therapy"[Mesh]) OR (((((Occupational Therapies[Title/Abstract]) OR (Therapies, Occupational[Title/Abstract])) OR (Therapy, Occupational[Title/Abstract])) OR (Ergotherapy[Title/Abstract])) OR (ergo therapies[Title/Abstract]))) AND (("Delirium"[Mesh]) OR (((((((Subacute Delirium[Title/Abstract]) OR (Delirium, Subacute[Title/Abstract])) OR (Deliriums, Subacute[Title/Abstract])) OR (Subacute Deliriums[Title/Abstract])) OR (Delirium of Mixed Origin[Title/Abstract])) OR (Mixed Origin Delirium[Title/Abstract])) OR (Mixed Origin Deliriums[Title/Abstract])))

15

**Web of Science Search Strategy (v0.1)**

# Database: All Databases

# Entitlements:

- WOS: 1985 to 2023

- KJD: 1980 to 2023

- MEDLINE: 1950 to 2023

- PPRN: 1991 to 2023

- PQDT: 1637 to 2023

- SCIELO: 2002 to 2023

# Searches:

1: (((((((TS=(Delirium)) OR TS=(Subacute Delirium)) OR TS=(Delirium, Subacute)) OR TS=(Deliriums, Subacute)) OR TS=(Subacute Deliriums)) OR TS=(Delirium of Mixed Origin)) OR TS=(Mixed Origin Delirium)) OR TS=(Mixed Origin Deliriums) Date Run: Sun Oct 15 2023 11:37:51 GMT+0800 (中国标准时间) Results: 33815

2: (((((TS=(Occupational Therapy)) OR TS=(Occupational Therapies)) OR TS=(Therapies, Occupational)) OR TS=(Therapy, Occupational)) OR TS=(Ergotherapy)) OR TS=(Ergotherapies) Date Run: Sun Oct 15 2023 11:39:35 GMT+0800 Results: 94215

3: #2 AND #1 Date Run: Sun Oct 15 2023 11:41:11 GMT+0800 Results: 146

**Embase**

Session Results

.......................................................

No. Query Results Results Date

#17. #7 AND #16 90 15 Oct 2023

#16. #8 OR #9 OR #10 OR #11 OR #12 OR #13 OR #14 OR 32,201 15 Oct 2023

#15

#15. 'mixed origin deliriums':ab,ti 15 Oct 2023

#14. 'mixed origin delirium':ab,ti 15 Oct 2023

#13. 'subacute deliriums':ab,ti 15 Oct 2023

#12. 'subacute deliriums':ab,ti 15 Oct 2023

#11. 'deliriums, subacute':ab,ti 15 Oct 2023

#10. 'delirium, subacute':ab,ti 2 15 Oct 2023

#9. 'subacute delirium':ab,ti 15 15 Oct 2023

#8. 'delirium':ab,ti 32,201 15 Oct 2023

#7. #1 OR #2 OR #3 OR #4 OR #5 OR #6 18,451 15 Oct 2023

#6. 'ergotherapies':ab,ti 15 Oct 2023

#5. 'ergotherapy':ab,ti 501 15 Oct 2023

#4. 'therapy, occupational':ab,ti 618 15 Oct 2023

#3. 'therapies, occupational':ab,ti 19 15 Oct 2023

#2. 'occupational therapies':ab,ti 165 15 Oct 2023

#1. 'occupational therapy':ab,ti 17,799 15 Oct 2023

.......................................................

**Cochrane 20231015_search_manager_search**

Search Name:

Date Run: 15/10/2023 12:03:19

Comment:

ID Search Hits

#1 (Occupational Therapy):ti,ab,kw OR (Occupational Therapies):ti,ab,kw OR (Therapies, Occupational):ti,ab,kw OR (Therapy, Occupational):ti,ab,kw OR (Ergotherapy):ti,ab,kw (Word variations have been searched) 7493

#2 (Ergotherapies):ti,ab,kw (Word variations have been searched) 31

#3 (Delirium):ti,ab,kw OR (Subacute Delirium):ti,ab,kw OR (Delirium, Subacute):ti,ab,kw OR (Deliriums, Subacute):ti,ab,kw OR (Subacute Deliriums):ti,ab,kw (Word variations have been searched) 5437

#4 (Delirium of Mixed Origin):ti,ab,kw OR (Mixed Origin Delirium):ti,ab,kw OR (Mixed Origin Deliriums):ti,ab,kw (Word variations have been searched) 7

#5 #1 OR #2 7493

#6 #3 OR #4 5437

#7 #5 AND #6 33

**Key word**

Occupational Therapy

Occupational Therapies

Therapies, Occupational

Therapy, Occupational

Ergotherapy

Ergotherapies

Delirium

Subacute Delirium

Delirium, Subacute

Deliriums, Subacute

Subacute Deliriums

Delirium of Mixed Origin

Mixed Origin Delirium

Mixed Origin Deliriums
